# Ischemic Stroke of Midbrain and Cerebellum Involving Reticular Activating System

**DOI:** 10.7759/cureus.1637

**Published:** 2017-09-01

**Authors:** Waleed Sadiq, Madeeha Subhan

**Affiliations:** 1 Department of Medicine, Shifa International Hospital; 2 Capital Hospital Islamabad, Ayub Teaching Hospital, Abbottabad

**Keywords:** midbrain, ascending reticular activating system, stroke

## Abstract

The reticular activating system is the part of the brain that maintains the sleep/wake cycle. Any damage to this region can cause hypersomnolence and drowsiness along with altered sensorium. This case presents a patient with cerebellar and midbrain stroke with infarct of the reticular activating system, leading to hypersomnolence, drowsiness, and altered sensorium.

## Introduction

Hypersomnia is defined as excessive daytime sleepiness, which may follow a stroke. Persistent hypersomnia was reported in only five percent of stroke patients [1]. Diffusion tensor tractography (DTT) has enabled a three-dimensional reconstruction and estimation of the activated reticular activating system in the live human brain [2]. The reticular acting system is a complex neural network connecting the reticular formation of the brainstem to the cerebral cortex through excitatory relays in the intralaminar nuclei of the thalamus. Assessing the ascending reticular activating system (RAS) is crucial in the diagnosis and management of patients with impaired consciousness [3]. Strokes of the midbrain can affect the RAS and lead to hypersomnia and cognitive impairment.

## Case presentation

A 67-year-old man with a medical history of hypertension and diabetes mellitus presented to the emergency department with altered sensorium and drowsiness for the prior six hours. He was talking with his wife when she noticed the symptoms. The patients had no loss of consciousness, headache, vomiting, urinary incontinence, or seizure before or after the symptoms started. His hypertension was diagnosed five years prior and was well controlled with angiotensin-converting enzyme inhibitors. His diabetes was controlled by insulin; his last recorded HbA1c was 6.9%. The patient reported that he is a smoker, and he is married with nine children. He avoids alcohol and eats a healthy diet. His family history is positive for stroke (his father). When he presented to the emergency department, his blood glucose level was 490 mg/dL, his blood pressure was 130/90 mmHg, his pulse was 81 bpm, and his respiratory rate was 19 breaths per minute. His Glasgow coma scale was 15/15, we noted horizontal nystagmus in both eyes, and the patient was drowsy. His gait was unsteady, and he was swaying towards the left side. He could not perform the heel-to-shin test on both sides, and his response to the finger-to-nose test was more affected on the left side. His reflexes were pendular in the lower extremities. We noted no facial asymmetry. The patient’s motor strength was 4/5 bilaterally in his upper and lower extremities. Sensations including fine touch, pain, vibration, and proprioception were intact. The patient’s response to the Babinski reflex test was normal. Based on these findings and unremarkable respiratory, cardiovascular, and gastrointestinal findings, we applied a stroke protocol, and the patient was started on regular insulin infusion, atorvastatin, aspirin, and we inserted a nasogastric tube. We chose to manage the patient conservatively. Laboratory evaluation results are presented in Table 1 and Table 2.

**Table 1:** Complete blood picture

ESR	108 mm/h
Hemoglobin	127 g/L
White blood cells	19.2 x x109/L
Red blood cells	4.2 x 1012/L
Platelets	326.2 x 109/L
MCV	82.4 fL

**Table 2:** Complete metabolic profile

Sodium	129 mmol/L
Potassium	3.9 mmol/L
Glucose	16.4 mmol/L
Total bilirubin	61.88 µmol/L
Alanine aminotransferase	0.566 µkat/L
Alkaline phosphatase	7.3 µkat/L
Urea nitrogen	7.1 mmol/L
Creatinine	97.2 µmol/L

**Figure 1:**
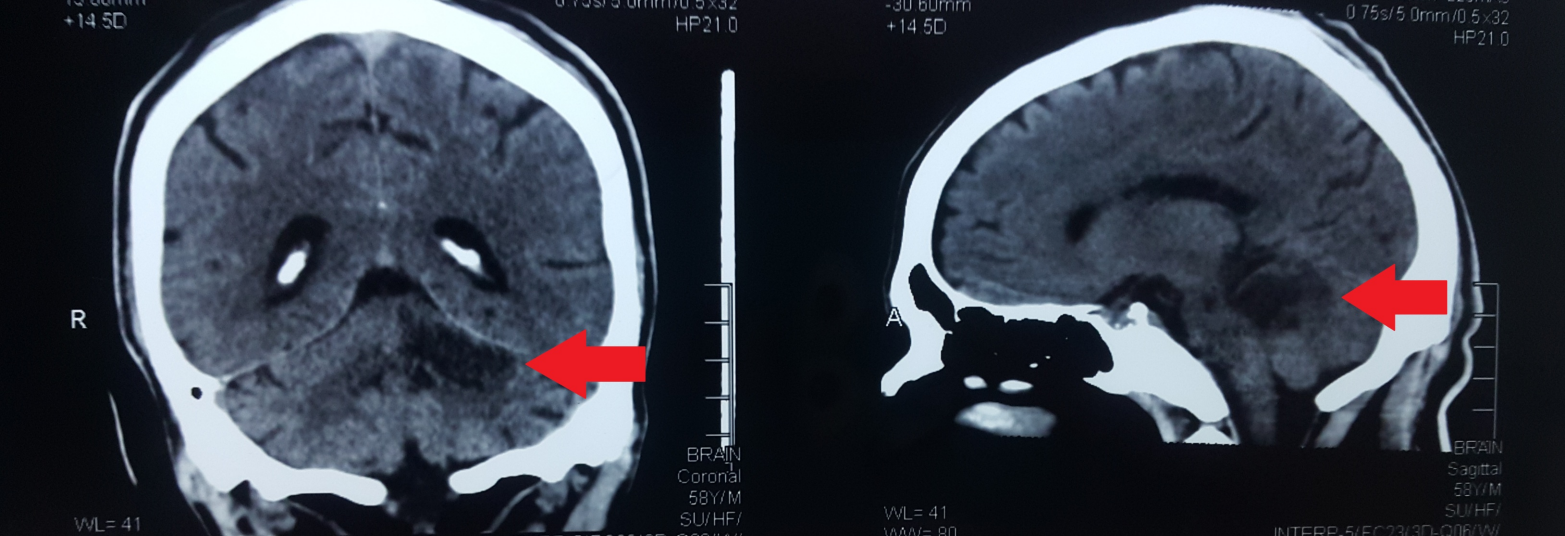
Ischemic infarct of left cerebellar hemisphere

**Figure 2:**
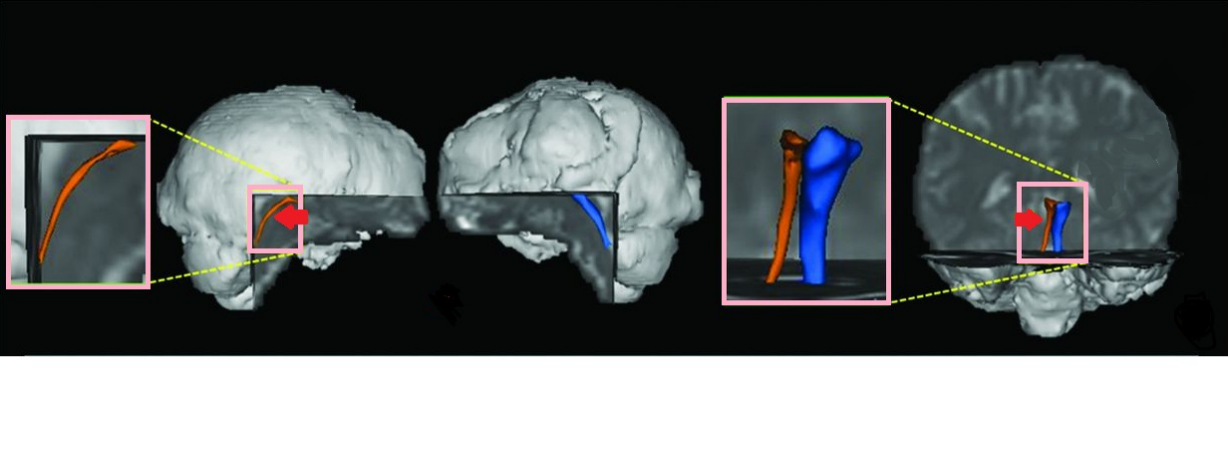
Diffusion tensor tractography showing thinning of the left lower reticular activating system

Radiological (computed tomography without contrast) findings are presented in Figure 1. We noted an effacement of cerebellar folia in a hypodense area of the hyperacute ischemic infarct of the left cerebellar hemisphere. We also noted a tiny lacunar infarct in the left basal ganglia and mild cerebral atrophy, as shown in Figure 1. Results of diffusion tensor tractography showing thinning of the left lower reticular activating system as compared to the right, as shown in Figure 2.

## Discussion

Stroke is a major cause of mortality and morbidity in both developing and developed countries. The RAS is associated with hypersomnia. A few studies using DTT have reported an association ascending RAS injury with hypersomnia [4-5]. To our knowledge, there have been few cases of cerebellar midbrain infarct involving the RAS causing drowsiness and hypersomnia. Many studies have suggested that the involvement of the ascending RAS might be the mechanism of hypersomnia in stroke patients. A case study ascribed narcolepsy to damage of the ventral ascending RAS in a patient with a traumatic brain injury [5]. Many studies have reported the hypothalamus is associated with hypersomnia; thus, the recovery of the patient's hypersomnia was due to the recovery of an injured lower ascending RAS [6]. As introduced by Moruzzi and Magoun in 1949, the basis of arousal in the brainstem has been linked to the ascending RAS [7]. The infarct of the midbrain and cerebellum involving the ascending RAS caused our patient to be hypersomnolent. During his stay and subsequent follow-up evaluations, the patient’s family reported that he was sleeping around 18 hours a day with an inability to perform the activities of daily living.

## Conclusions

Our results suggest that patients presenting with nystagmus, hypersomnolence, and drowsiness must be assessed for RAS damage along with cerebellar and midbrain damage. Given this study is a case report, further studies with larger patient samples are necessary to validate our findings. In addition, the critical region of the RAS for hypersomnolence and drowsiness should be studied further. Therefore, any stroke patient presenting with altered sensorium, drowsiness, and sleeping while talking should undergo DTT to assess the extent of RAS damage.
